# Swelling and Collapse of Cylindrical Polyelectrolyte Microgels

**DOI:** 10.3390/polym14225031

**Published:** 2022-11-20

**Authors:** Ivan V. Portnov, Alexandra A. Larina, Rustam A. Gumerov, Igor I. Potemkin

**Affiliations:** 1 Physics Department, Lomonosov Moscow State University, 119991 Moscow, Russia; 2 A. N. Nesmeyanov Institute of Organoelement Compounds, Russian Academy of Sciences, 119991 Moscow, Russia; 3 National Research South Ural State University, 454080 Chelyabinsk, Russia

**Keywords:** cylindrical microgels, collapse, electrostatics, computer simulations

## Abstract

In this study, we propose computer simulations of charged cylindrical microgels. The effects of cross-linking density, aspect ratio, and fraction of charged groups on the microgel swelling and collapse with a variation in the solvent quality were studied. The results were compared with those obtained for equivalent neutral cylindrical microgels. The study demonstrated that microgels’ degree of swelling strongly depends on the fraction of charged groups. Polyelectrolyte microgels under adequate solvent conditions are characterized by a larger length and thickness than their neutral analogues: the higher the fraction of charged groups, the longer their length and greater their thickness. Microgels’ collapse upon solvent quality decline is characterized by a decrease in length and non-monotonous behavior of its thickness. First, the thickness decreases due to the attraction of monomer units (beads) upon collapse. The further thickness increase is related to the surface tension, which tends to reduce the anisotropy of collapsed objects (the minimum surface energy is known to be achieved for the spherical objects). This reduction is opposed by the network elasticity. The microgels with a low cross-linking density and/or a low enough aspect ratio reveal a cylinder-to-sphere collapse. Otherwise, the cylindrical shape is preserved in the course of the collapse. Aspect ratio as a function of the solvent quality (interaction parameter) demonstrates the maximum, which is solely due to the electrostatics. Finally, we plotted radial concentration profiles for network segments, their charged groups, and counterions.

## 1. Introduction

Polymer microgels are soft, porous, colloidally stable macromolecular objects that reveal the properties of polymers, (nano-)microparticles, and surfactants [1]. They have a network-like internal structure and their size ranges between tens of nanometers and tens of microns (the upper limit is determined by stability towards the precipitation of the single macromolecules). Such as for hydrogels, the most spectacular property of the microgels is their ability to drastically swell and collapse under external stimuli, e.g., temperature [2], pH [3], magnetic field [4], etc., and significantly change their size, porosity, and characteristics of their interaction with each other (from repulsion in the swollen state to attraction in the collapsed one). However, in contrast to the macroscopic gels, the microgels’ stimuli response is considerably faster and makes them very promising for many applications. In particular, they can be used as carriers for guest molecules, which can be released on demand [5,6]; as porous and functional alternatives to solid particles for emulsion stabilization [7,8]; in catalysis [9,10]; as scavengers [11]; in membrane technologies [12]; in tissue engineering [13]; and many others.

The common methods of microgel synthesis include precipitation [14], (mini)emulsion, and [15] template [16] polymerization, as well as microfluidics-based synthesis. [17] In the case of precipitation and (mini)emulsion polymerization the obtained microgels are spherical in shape due to the presence of surface tension, the minimum of which is only achieved in the spherical geometry. Indeed, the growing chains in precipitation polymerization are surrounded by a poor quality solvent while microgels in (mini)emulsion polymerization reproduce the shape of the droplets that also have surface tension and a spherical shape [18]. On the contrary, microfluidics provides control of the microgel shape, varying it from spherical to cylindrical, and the latter’s aspect ratio is controlled by droplet volume with respect to the diameter of the channel [19]. More sophisticated shapes, including an anisotropic core-shell or hollow [20,21,22], ribbon-like microgels, can be obtained via template polymerization [23]. However, a pretty large size in some cases could be considered a disadvantage of this technique. The design of nanoscale anisotropic microgels is still challenging.

Given that the properties of the spherical microgels are studied well enough [1], much less is known about anisotropic microgels [21,22]. In particular, it was recently found that anisotropic microgels adsorbed at oil–water and air–water interfaces upon compression demonstrate self-assembly into liquid-crystalline and more sophisticated structures, caused by excluded volume and capillary forces [22]. The incorporation of magnetic nanoparticles into anisotropic microgels can lead to monovalent cationic charged groups of the microgel being randomly distributed over the network. The same amount of counterions is added to the system to provide a macroscopic electric neutrality of the system. The fraction of charged groups *f* was varied between 0 and 50%. Note that such high charge content can be obtained in reality, for instance, via their previous orientation in the magnetic field, forming a strongly anisotropic medium, which is a perspective for directed cell growth [13,24]. Liquid-crystalline ordering in solutions of the cylindrical microgels is predicted for their high enough concentrations depending on their aspect ratio and cross-linking density [25]. The swelling and collapse of neutral cylindrical microgels demonstrates peculiar behavior. Depending on microgels’ cross-linking density, aspect ratio, and molecular weight, one can observe either self-similar (cylinder-to-cylinder) or non-self-similar collapse, leading to cylinder-to-sphere transformation [25]. Therefore, in addition to microgel size and porosity, their aspect ratio in the solution and self-assembled structures can be finely tuned by external stimuli.

In this work, we continued the study of the swelling and collapse of the cylindrical microgels with computer simulations. The main aim was to reveal the effect of electrostatics on the swelling and collapse of the single microgels. Both effects of cross-linking density and fraction of charged groups were investigated. We demonstrate how microgels’ length and thickness (as well as aspect ratio) change upon solvent quality decline (temperature increase for thermoresponsive gels). A comparison with equivalent neutral microgels is provided here. The radial concentration profiles of monomer units and charged groups were analyzed. The effect of the fraction of charged groups on the distribution of counterions are demonstrated here.

## 2. Model and Simulation Methods

We have used a conventional Brownian dynamics technique of simulations within a coarse-grained model and with an implicit solvent. All structural units of the microgel (charged and neutral monomer units) and counterions are modeled as beads of equal radius σ and mass *m*, interacting with each other through the truncated Lennard-Jones potential [26]:

(1) $$U_{LJ}=4\varepsilon \left[{\left(\sigma /r_{ij}\right)}^{12}-{\left(\sigma /r_{ij}\right)}^{6}\right],\;\;r_{ij}\;\leq r_{cut},$$

where *r_ij_* is the distance between two interacting beads and *r_cut_* is a cut-off radius beyond which the potential is equal to zero. The cut-off radii for interaction of the monomer units with each other and counterions with the monomer units and each other were selected as *r_cut_* = 2.5 σ and *r_cut_* = 2^1/6^ σ, respectively. The solvent quality is determined by the interaction parameter ε of the potential acting between microgel beads. The increase in this parameter corresponds to the solvent quality decline. In our simulations, ε varies between 0.01 *k_B_T* (favorable solvent quality) and 1.4 *k_B_T* (poor solvent quality) [27]. A similar parameter describing interactions of the counterions with each other and the monomer units was fixed to the value 1 *k_B_T* in all simulations. Electrostatic interactions between any pair of the charged species were described by Coulomb potential. The Bjerrum length was chosen as *l*_B_ = 1 σ and corresponds to the aqueous solutions [28,29].

**Figure 1:**
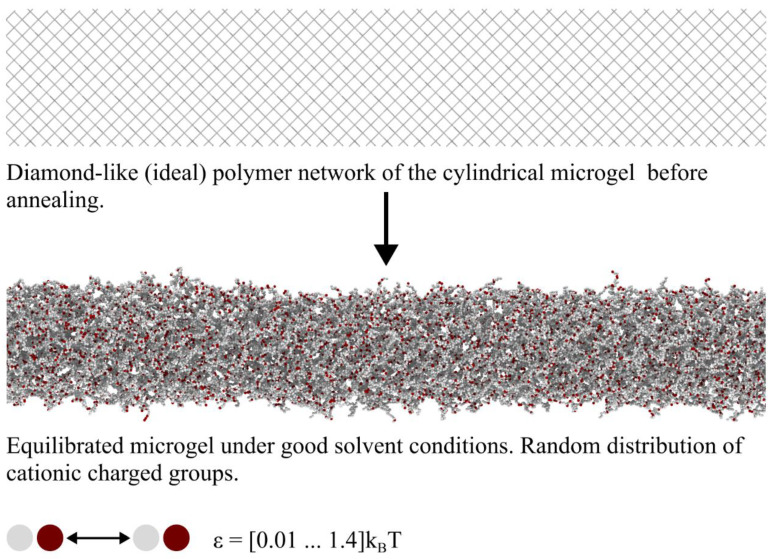
Internal structure (thin layer near the axis) of the cylindrical microgel before and after annealing. The charged groups (red beads) are randomly distributed over the network. Interactions between network beads are quantified by the interaction parameter ε.

The cylindrical microgels were designed as follows. Fully stretched subchains of an ideal microgel (all subchains have equal length) were connected through tetrafunctional cross-links in such a way that repeats a unit cell of the diamond crystal lattice. Then, a parallelepiped microgel template consisting of 10 × 10 × 100 modified unit cells, mentioned above, was constructed. The subchain length *M* was considered to be 10 and 20 corresponding to approximately 5% and 2.5% of cross-links, respectively. To provide a cylindrical shape to the microgel particles, a cylinder was inscribed into the template in such a way that the cylinder axis passes through a set of the cross-links belonging to a straight. Then, all beads outside the cylinder were “cut off” (Figure 1). The cylinder is characterized by the initial aspect ratio *A*, i.e., the ratio of the length *L_0_* to its diameter 2*R_0_*, and *A* = *L_0_*/2*R_0_* under preparation conditions. Annealing of the microgel and a change in the solvent quality can lead to the change in the aspect ratio. Two aspect ratio values, *A* = 4 and 10, were considered. The number of beads (the molecular weight) in the different microgels *N* was fixed at 50 and 100 thousand.

Monovalent cationic charged groups of the microgel are randomly distributed over the network (Figure 1). The same amount of counterions is added to the system to provide a macroscopic electric neutrality of the system. The fraction of charged groups f was varied between 0 and 50%. Note that such high charge content can be obtained in a reality, for instance, via the post modification of network monomers [30]. adjacent bonded beads of a chain are connected by a finitely extensively, nonlinear, elastic (FENE) spring potential: [31]

(2) $$U_{FENE}=-\frac{k}{2}R_{0}^{2}\ln\left[1-{\left(\frac{r}{R_{0}}\right)}^{2}\right],\;r<R_{0}$$

with parameters *k* = 30 *k_B_T*/σ^2^ and *R*_0_ = 1.5 σ. The simulations were performed using the open-source software LAMMPS [32] in NVT ensemble with imposed periodic boundary conditions; the linear sizes of the simulation box were *L_y_* = *L_z_* = 500 σ and *L_z_* = 1500 σ. The electrostatic interactions between charged beads were calculated using the PPPM algorithm [33] with an accuracy of 10^−5^. The equations of motion were integrated with a time step of Δt = 0.005 τ, where τ is the standard time unit for a Lennard-Jones fluid. Initially, single microgels with fully stretched chains and counterions uniformly distributed throughout the box were annealed (equilibrated) for 8 × 10^6^ steps under adequate solvent conditions, ε = 0.01 *kT*. The statistics were gathered within additional 2 × 10^6^ steps. Then, the interaction parameter was increased until the value ε = 1.4 *kT* was reached. For each case of solvent quality, the amount of simulation time (equilibration + statistics) was the same.

## 3. Results and Discussion

**Figure 2:**
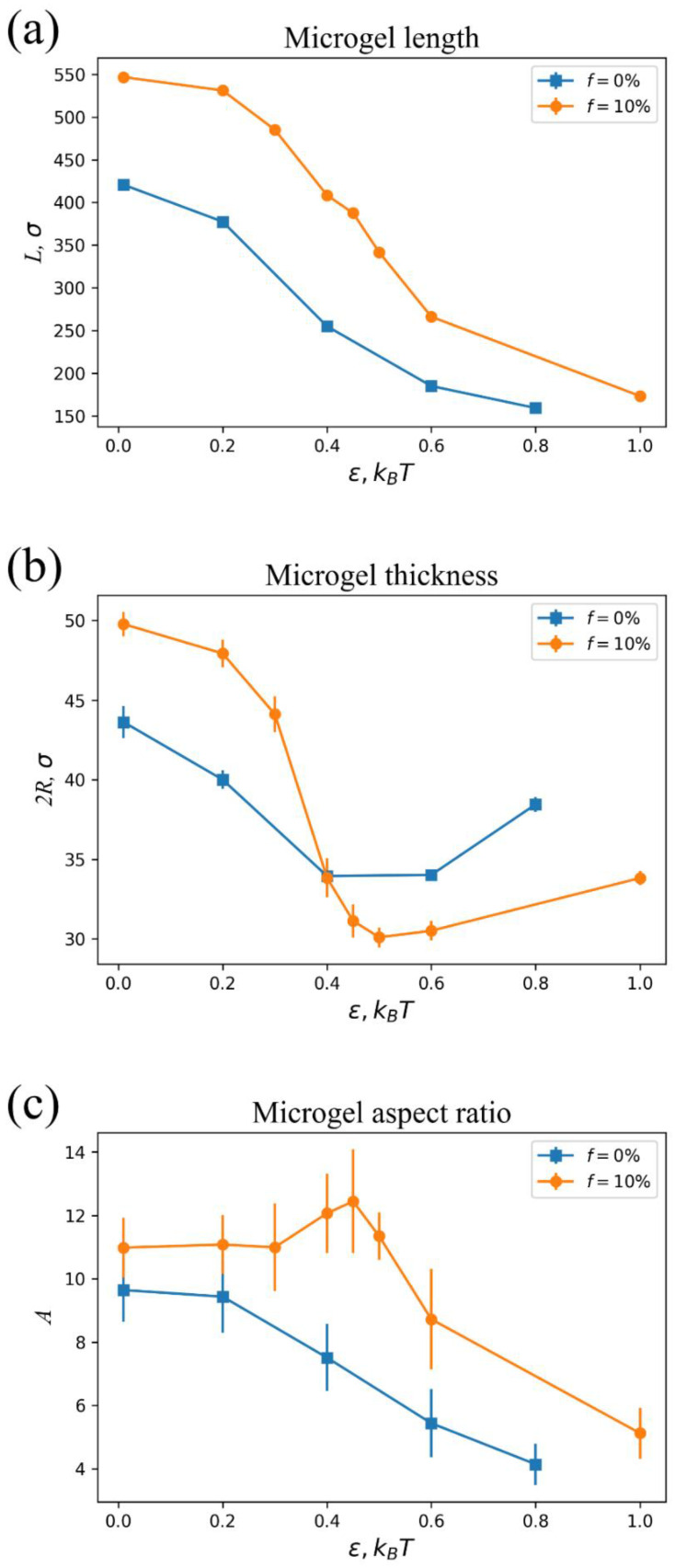
Contour length *L* (**a**), thickness 2*R* (**b**), and aspect ratio *A* = *L*/2*R* (**c**) of equivalent neutral (*f* = 0%) and charged (*f* = 10%) microgels as functions of the interaction parameter ε. The molecular weight and subchain length of the microgels are *N* = 100,000 and *M* = 10, respectively. The initial aspect ratio of the microgels is *A* = 10.

**Figure 3:**
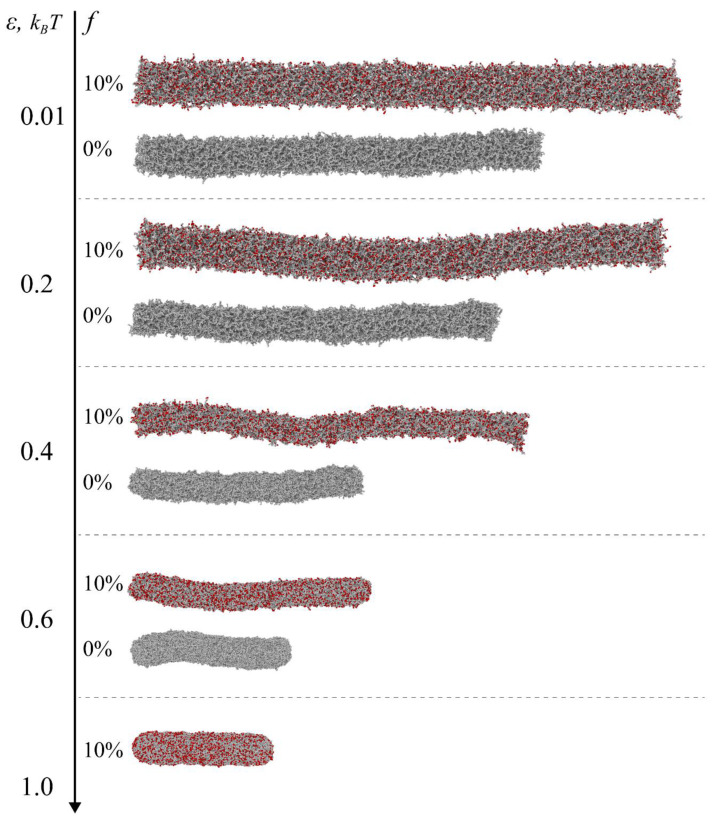
Side-view snapshots of equivalent neutral (*f* = 0%) and charged (*f* = 10%) microgels upon solvent quality decline. The charged groups were slightly enlarged for clarity. The molecular weight and subchain length of the microgel are *N* = 100,000 and *M* = 10, respectively. The initial aspect ratio of the microgels is *A* = 10.

First, let us demonstrate how the presence of charges affects the swelling behavior of the cylindrical microgels. In our research, interaction parameter ε describes the attraction between microgel segments and quantifies solvent quality: the larger the parameter, the poorer the solvent quality. Figure 2a,b shows contour length *L* and thickness 2*R* of single charged and neutral microgels as functions of ε. The contour length is determined as the sum of distances between neighbor cross-links, which the cylinder axis passes through under the preparation conditions [25]. It is nearly equal to the apparent length of the cylindrical microgels. In turn, the thickness (the diameter of a cylinder) is determined geometrically from a slab of a network cut from its central part (determined from a center of mass) with a length equal to 20% of *L*. Figure 2a clearly demonstrates that the presence of charged groups leads to a significant elongation of the microgel as compared to the neutral one. This effect is due to the osmotic pressure of those counterions, which are localized within the microgel, and electrostatic repulsion of similarly charged groups of the network. In turn, the latter is due to the local violation of the electric neutrality of the microgel caused by the partial release of the counterions [27,34,35] (see below). Both neutral and charged microgels shorten upon the worsening of the solvent quality. Such a behavior is caused by the attraction of neutral beads, leading to the microgel collapse. However, a charged microgel always has a longer length than a neutral one under the same value of the parameter ε (the same solvent quality). Equal lengths of the neutral and charged microgels can be achieved upon the stronger attraction of the beads in the charged microgel to overcome the exerted osmotic pressure of counterions and electrostatic repulsion. At the same time, the more interesting behavior is found for microgel thickness (Figure 2b). Under favorable solvent conditions, ε = 0.01 *kT*, the polyelectrolyte network is thicker than the neutral one and, keeping in mind the same ratio for the length, we can say that the charged microgel is more swollen than the equivalent neutral one. Such an effect is well-known for spherical microgels [36,37] and macroscopic gels [38]. Initially, the thickness decreases with an increase in ε for both neutral and charged networks (Figure 2b). This decrease is caused by microgel collapse, leading to the increase in polymer concentration within the networks (see below). However, a further increase in ε is responsible for microgel thickening (in both cases (Figure 2b)), which is also visible in the snapshots in Figure 3. This behavior is a feature of a finite-sized anisotropic object and is caused by the presence of surface tension. Indeed, if microgels did not have elasticity, an increase in surface tension (solvent quality decline) would lead to the transformation of the cylinder to the sphere because the latter has a smaller surface area than the former (under fixed volume). Therefore, in the presence of microgel elasticity, the cylinder shortening and thickening at high ε values is driven by surface tension. In contrast to the *L*(ε) dependences (Figure 2a), 2*R*(ε) curves cross each other (Figure 2b). This means that, at a certain solvent quality, the thickness of the charged microgel can be less than that of the neutral one. This effect is a consequence of the anisotropy of the object and long-range electrostatic repulsion between the network’s charged groups. An increase in polymer concentration within the charged microgel upon collapse proceeds via microgel shortening and thinning. However, thinning is electrostatically more favorable than shortening. Indeed, the electrostatic energy of a cylindrical object is proportional to *Q*^2^/*L,* apart from a logarithmic factor [39]. Here, *Q* and *L* are a charge and a length of a cylinder, respectively. Therefore, the electrostatic energy decreases upon elongation (such as in the case of linear polyelectrolyte chains in dilute solution) [40]. That is why microgel length can less progressively decrease than thickness upon collapse. This behavior is detectable if we plot the aspect ratio function (Figure 2c). The *A*(ε) dependence is non-monotonous for polyelectrolyte microgels. The initial increase in *A* is caused by electrostatics: the surface tension is too low to overcome the favorable electrostatic energy for an elongated object. At higher ε values, the aspect ratio decreases, meaning that surface tension dominates electrostatics (Figure 2c). Meanwhile, for the neutral microgel, *A* monotonously decreases with ε (Figure 2c).

**Figure 4:**
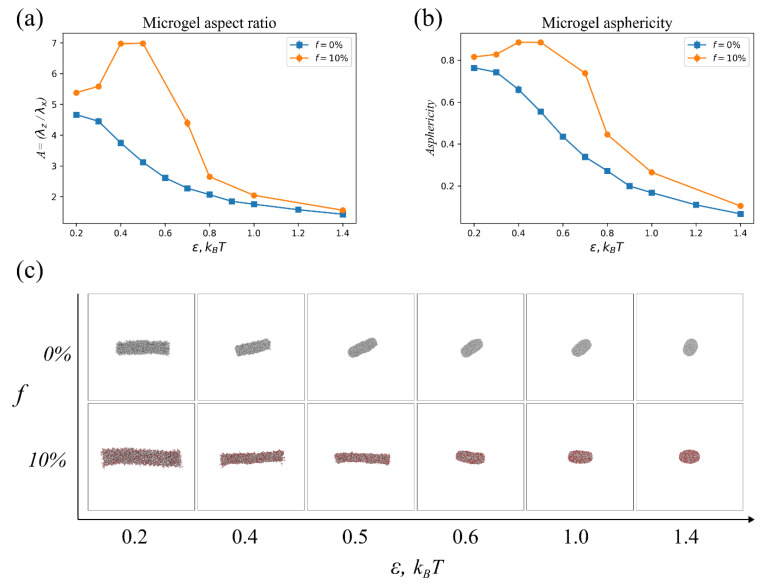
(**a**) Aspect ratio calculated from eigenvalues of gyration tensor *λ_z_*/*λ_x_* [41]. (**b**) Asphericity parameter and (**c**) snapshots of equivalent neutral (*f* = 0%) and charged (*f* = 10%) microgels vs. interaction parameter ε. The molecular weight and subchain length of the microgel are *N* = 50,000 and *M* = 10, respectively. The initial aspect ratio of the microgels is *A* = 4.

Despite the difference in collapse behavior, the considered neutral and charged microgels are common in a sense of so-called “rod-to-rod” collapse: the final (under poor quality solvent conditions) values of the aspect ratio *A* > 4 (Figure 2c). On the contrary, a near “rod-to-sphere” transition is observed for both microgels if we take a shorter and smaller cylinder. Figure 4 demonstrates the transition for the microgels with the initial (under preparation condition) aspect ratio *A* = 4, molecular weight *N* = 50,000, and the same subchain length *M* = 10. For convenience, the *A* parameter is determined from the eigenvalues of a gyration tensor [41]. While for longer cylinders, this approach produces similar values as from the ratio of contour length and diameter, for shorter and smaller cylinders, it allows for more apparent tracking size changes.

Under adequate solvent conditions, ε = 0.01 *kT*, both microgels are swollen, revealing a cylindrical shape, and the charged sample has a longer length and greater thickness (Figure 4c). A collapse of the neutral microgel is characterized by a more progressive shortening with respect to the thinning: the aspect ratio monotonously decreases with ε (Figure 4a). Such as for longer counterparts, the charged microgel first decreases thickness upon a more progressive collapse, which corresponds to the aspect ratio increase (Figure 4a). This effect also occurs due to the electrostatics, which stabilizes the elongated shape. However, the further solvent quality decline (an increase in ε) leads to the formation of a nearly spherical microgel. Furthermore, conformations of neutral and charged microgels are nearly equal at highest considered values of ε (Figure 4c). The aspect ratio curves for different microgels converge at these values as well. An asphericity parameter [41] shown in Figure 4b demonstrates the evolution of the microgels’ shape: cylindrical and spherical shapes are quantified by the values > 0.6 and <0.1, respectively.

**Figure 5:**
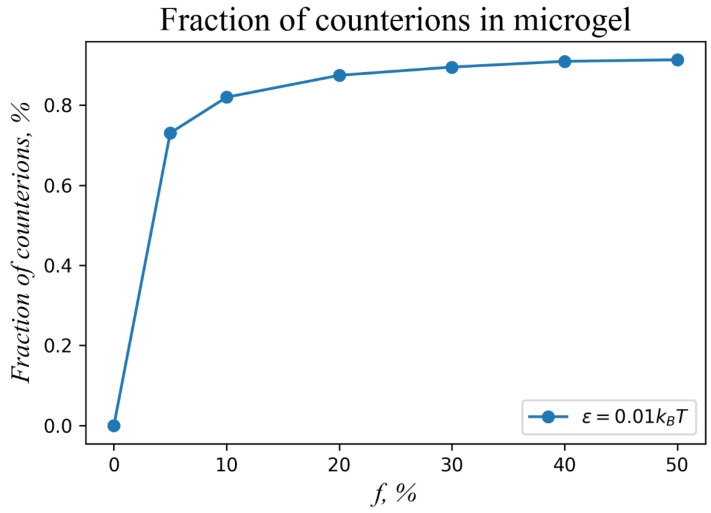
Fraction of counterions localized within the charged microgel as a function of the fraction of charged groups of the network *f*. The molecular weight and subchain length of the microgel are *N* = 100,000 and *M* = 10, respectively. The initial aspect ratio is *A* = 10. The microgel is modeled under favorable solvent conditions, ε = 0.01*kT*.

The non-monotonous dependence of the aspect ratio on the solvent quality is explained by the presence of the uncompensated charge of the microgel. To prove this hypothesis, we plot a dependence of the fraction of counterions *β*, which are localized within the microgel, on the fraction of charged groups *f* in the network (Figure 5). It can be seen that the fraction of localized counterions depends on the fraction of the charged groups: the higher the *f* value, the larger the fraction of localized counterions. However, it does not mean that more charged microgels have lower net charge. On the contrary, the microgel net charge fraction increases with *f* due to the convex shape of the *β*-*f* dependence, *β* ~ *f^α^*, *α* < 1. Indeed, if the microgel total net charge is introduced as *Q*, its ratio to the total number of beads (relative net charge) in the network *N* would take the form *Q/N = ef*(1-*β*), where *e* is the elementary charge. Thus, for the 10%, 20%, and 40% microgels, the relative net charge grows as *Q/Ne ≈* 1.8%, 2.5%, 3.6%, respectively (Figure 5).

**Figure 6:**
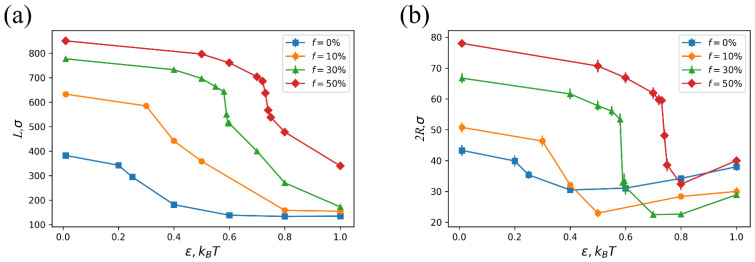
Microgel contour length *L* (**a**) and thickness 2*R* (**b**) vs. interaction parameter ε between beads. Different curves correspond to the different fraction of charged groups in the microgel. The molecular weight and subchain length of the microgel are *N* = 50,000 and *M* = 20, respectively. The initial aspect ratio is *A* = 10.

The effect of the fraction of charged groups in the network on microgel collapse is demonstrated in Figure 6. The microgel degree of swelling under favorable solvent conditions strongly depends on the fraction of charged groups. Both the microgel contour length and thickness increase with *f* (Figure 6a,b, respectively). This is mainly due to the increase in the number of counterions, which creates exerted osmotic pressure. Additionally, for this reason, stronger attraction between monomer units is required to induce microgel collapse: the transition to the collapsed state shifts towards higher ε values (Figure 6). We can see that the collapse becomes more abrupt upon the increase in the fraction of charged groups. This effect is an indication of the polyelectrolyte nature: the collapse of macroscopic gels (a limiting case of an infinitely sized network) proceeds as a first order phase transition [42].

**Figure 7:**
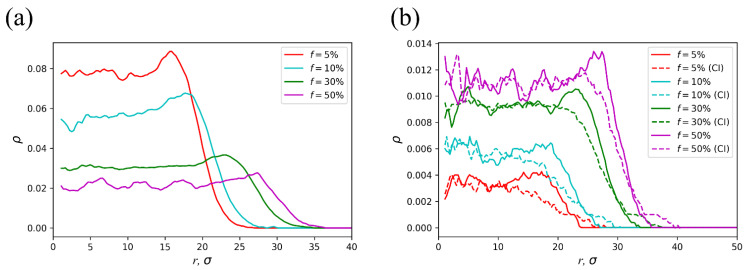
(**a**) Polymer volume fraction, (**b**) volume fraction of the network charged groups (solid) and counterions (dashed) as functions of the radial coordinate *r* at different *f* values and favorable solvent conditions, ε = 0.01 *kT*. The molecular weight and subchain length of the microgel are *N* = 100,000 and *M* = 10, respectively. The initial aspect ratio is *A* = 10.

The radial concentration profiles for microgels differing in the fraction of charged groups under adequate solvent conditions are presented in Figure 7. They were obtained as follows. The microgel was divided into a few cylindrical segments of the length *l* = 0.2*L* (similar to the determination of microgel thickness). The concentration profiles were calculated for each inner segment (excluding end segments) with further averaging over the segments and over their rotation with respect to the axis. The polymer volume fraction (dimensionless concentration of polymer beads) is presented in Figure 7a. The highest swelling of the microgel is characterized by the lowest polymer concentration and was achieved for *f* = 50%. The decrease in *f* leads to the increase in the polymer concentration within the microgel (Figure 7a). We can also observe that the polymer volume fraction has a local maximum at the microgel periphery. This maximum is more distinct for the lowest fraction of charged groups and diminishes upon microgel charging (Figure 7a). Such an effect is known for the spherical microgels and is related to the partial redistribution of the charged groups towards the periphery to reduce the electrostatic energy [27]. The distribution of the charged groups of the network and counterions is presented in Figure 7b by solid and dashed lines, respectively. For all considered *f* values, we observed the local increase in the fraction of the network charged groups at the periphery. Here, we can also see a maximum violation of the electric neutrality: the counterion concentration profiles significantly deviate from those of the network charged groups at the microgel periphery. This is caused by the entropy of counterions. Therefore, at the periphery, an electric double layer is formed.

**Figure 8:**
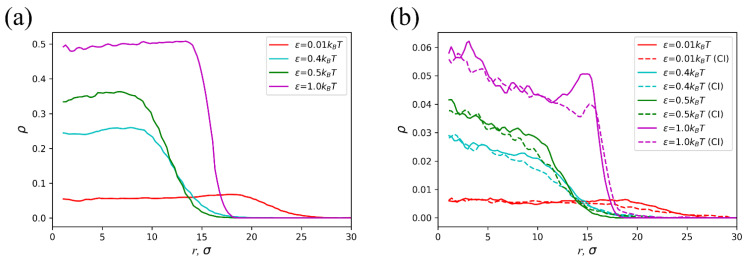
(**a**) Polymer volume fraction, (**b**) volume fraction of the network charged groups (solid) and counterions (dashed) as functions of the radial coordinate *r* at different ε values. The molecular weight and subchain length of the microgel are *N* = 100,000 and *M* = 10, respectively. The initial aspect ratio and the fraction of charged groups are *A* = 10 and *f* = 10%.

The evolution of the polymer concentration profile and the positions of charged groups and counterions upon solvent quality decline are presented in Figure 8. The polymer concentration gradually increases in the microgel interior with ε. Such as under favorable solvent conditions, a maximum concentration value is detectable at the periphery in a poor quality solvent, although the difference with respect to the inner part is not essential (Figure 8a). This effect is due to the violation of the electric neutrality in the peripheral layer, which therefore leads to a lower concentration of counterions and, further, to less exerted osmotic pressure (Figure 8b). Indeed, counterion concentration in the microgel interior is higher and gradually decreases towards the periphery (Figure 8b).

## 4. Conclusions

In this study, we performed computer simulations of the swelling and collapse of cylindrical microgels bearing charged groups. Both aspect ratio and charged group fraction effects on microgel collapse were studied. A comparison with neutral counterparts is provided here. We have shown that under adequate solvent conditions, charged microgels have a longer length and greater thickness than their neutral counterparts. This effect primarily occurs due to the exerted osmotic pressure of counterions localized within the microgel to compensate for the electric charge of the network. Such as for macroscopic polyelectrolyte gels, the swelling degree of the cylindrical microgel depends on the fraction of charged groups: the higher the fraction, the greater the swelling (length and thickness of the microgel). We detected peculiarities in the microgel collapse upon solvent quality decline. An increase in the attraction between beads (interaction parameter ε) leads to microgel shortening. However, the microgel thickness demonstrates non-monotonous behavior. The initial decrease caused by the attraction between beads is followed by the microgel thickening, which is a consequence of the surface tension. Though, if it has a lower value for the less anisotropic object, the microgel would collapse into the sphere in the absence of elasticity. In addition, in the case of low cross-linking density or a low enough aspect ratio, we observed a “cylinder-to-sphere” collapse. Otherwise, the cylindrical shape is preserved upon collapse. The microgel aspect ratio (the length-to-diameter ratio) has a maximum in the course of collapse. The initial growth (more progressive thinning with respect to the shortening) is caused by electrostatics. The microgel releases some counterions due to entropic reasons and has uncompensated charge. The elongated charged objects have less electrostatic energy than their spherical counterparts (similar to polyelectrolyte chains in a dilute solution). Therefore, the microgel collapse accompanied by thinning is more favorable. The further decrease in the aspect ratio is driven by the surface tension. We have demonstrated that the characteristic of the collapse depends on the fraction of charged groups: the higher the fraction, the more abrupt the transition. The computer simulations allowed us to plot polymer concentration profiles and demonstrate the distribution of charged groups and counterions. Because of the relative novelty of the technique for synthesizing microgels of complex architectures [16,23,24], and so far, because of the current absence of methods of their preparation on a nanometer scale, there is little information in the literature on the experiments with anisotropic polyelectrolyte networks. Therefore, our research has both fundamental and practical significance. We believe that it can inspire the creation of functional materials with specified properties based on cylindrical microgels.

## Data Availability

The data presented in this study are available on request from the corresponding author.
